# Lurcher Mouse as a Model of Cerebellar Syndromes

**DOI:** 10.1007/s12311-025-01810-5

**Published:** 2025-02-28

**Authors:** Nilpawan Roy Choudhury, Pascal Hilber, Jan Cendelin

**Affiliations:** 1 Department of Pathological Physiology, Faculty of Medicine in Pilsen, Charles University, Pilsen, Czech Republic; 2 Univ Rouen Normandie, Inserm, Normandie Univ, CBG UMR 1245 NeuroGlio Team, Rouen, France; 3 Institute of Research and Innovation in Biomedicine (IRIB), Rouen, 76000 France; 4 Laboratory of Neurodegenerative Disorders, Biomedical Center, Faculty of Medicine in Pilsen, Charles University, Pilsen, Czech Republic; 5 Department of Pathophysiology, Faculty of Medicine in Pilsen, Charles University, alej Svobody 76, Plzen, 323 00 Czech Republic

**Keywords:** Ataxia, Cerebellum, Lurcher Mouse, Cerebellar Cognitive Affective Syndrome, Validity

## Abstract

Cerebellar extinction lesions can manifest themselves with cerebellar motor and cerebellar cognitive affective syndromes. For investigation of the functions of the cerebellum and the pathogenesis of cerebellar diseases, particularly hereditary neurodegenerative cerebellar ataxias, various cerebellar mutant mice are used. The Lurcher mouse is a model of selective olivocerebellar degeneration with early onset and rapid progress. These mice show both motor deficits as well as cognitive and behavioral changes i.e., pathological phenotype in the functional domains affected in cerebellar patients. Therefore, Lurcher mice might be considered as a tool to investigate the mechanisms of functional impairments caused by cerebellar degenerative diseases. There are, however, limitations due to the particular features of the neurodegenerative process and a lack of possibilities to examine some processes in mice. The main advantage of Lurcher mice would be the expected absence of significant neuropathologies outside the olivocerebellar system that modify the complex behavioral phenotype in less selective models. However, detailed examinations and further thorough validation of the model are needed to verify this assumption.

## Clinical Trial Number

Not applicable.

## Introduction

The cerebellum is known to play a role in a broad spectrum of brain functions [1–3]. Cerebellar diseases are manifested not only by the cerebellar motor syndrome including cerebellar ataxia, intention tremor, and passivity [4], but also in some cases by cerebellar cognitive affective syndrome (CCAS), also referred to as Schmahmann’s syndrome [4–6]. However, the mechanisms of the functional changes are not clear. It is not always known whether they are due to the direct involvement of the cerebellum in the given function, secondary changes in the brain, or indirect cross-effects between various functions. The performance of both patients and cerebellar animal models in various examinations and functional tests can be influenced by multiple factors. For instance, performance in motor tests can be modified by motivation. And vice versa, cognitive and behavioral tests might be influenced by motor performance or sensory disturbances [7, 8].

CCAS is defined as a neuropsychiatric manifestation of cerebellar dysfunction [5, 6]. It has multiple etiologies and mechanisms of cerebellar tissue (or cerebellar function) damage. The wide spectrum of diseases and noxious factors affecting the cerebellum might not only have diverse consequences on the cerebellum as such, but also potential extracerebellar (even extra neural) impacts on the organism, complicating the attribution of individual signs specifically to cerebellar dysfunction [6, 9–12]. It might modify the resulting pathological phenotype on the functional level, i.e., neurological and psychiatric manifestations [6, 9, 10, 12]. Therefore, animal models of cerebellar diseases cannot be of universal applicability, but we must consider etiology and pathogenesis. For instance, mechanical destruction of the cerebellum is quite different from severe cerebellar degeneration, although in both cases there could be a substantial loss of cerebellar functional capacity and cerebellar dysfunction manifested as cerebellar motor syndrome and/or CCAS as the underlying issues [6, 11]. Among human hereditary cerebellar degenerations, there is a high diversity of pathogenesis and manifestations, many of which remain incompletely understood [13, 14]. This diversity is also reflected in the diversity of the mouse models of these diseases. Mutants such as staggerer, reeler, and hot-foot, with specific cerebellar lesions, are valuable tools for investigating cerebellar diseases.

Among all these cerebellar mutant models, the Lurcher mouse, discovered in the Medical Research Council Radiobiological Research Unit at Harwell, England, in 1954 [15] is still of great interest. In this review, we firstly summarize the knowledge about Lurcher mutant mice, with special emphasis on their cognitive and behavioral disturbances and thereafter we discuss the potential validity of this mutant as an animal model of CCAS.

## The δ2 Glutamate Receptor

Lurcher pathology is caused by mutation in the *GluRδ2* gene encoding the mouse δ2 glutamate receptor (GluRδ2, also known as the GluD2 receptor or GluRD2), located on chromosome 6 [16, 17].

In the healthy state, GluRδ2 is an ionotropic glutamate receptor (iGluR), predominantly expressed in Purkinje cells (PCs) [18], as well as at lower levels in various other brain areas, including the cerebral cortex, hippocampus, thalamus, striatum, mesencephalon, and olfactory bulb [19]. Although the name suggests a connection to the glutamate receptor, glycine and D-serine, rather than the glutamate neurotransmitter, act as agonists of GluRδ2 [20, 21]. GluRδ2 was included in the iGluR family, as its structure resembles the other three classes (α-amino-3-hydroxy-5-methyl-4-isoxazolepropionic acid receptor - AMPAR, N-methyl-D-aspartate receptor NMDAR, and Kainate receptor) of that family [22].

**Fig. 1:**
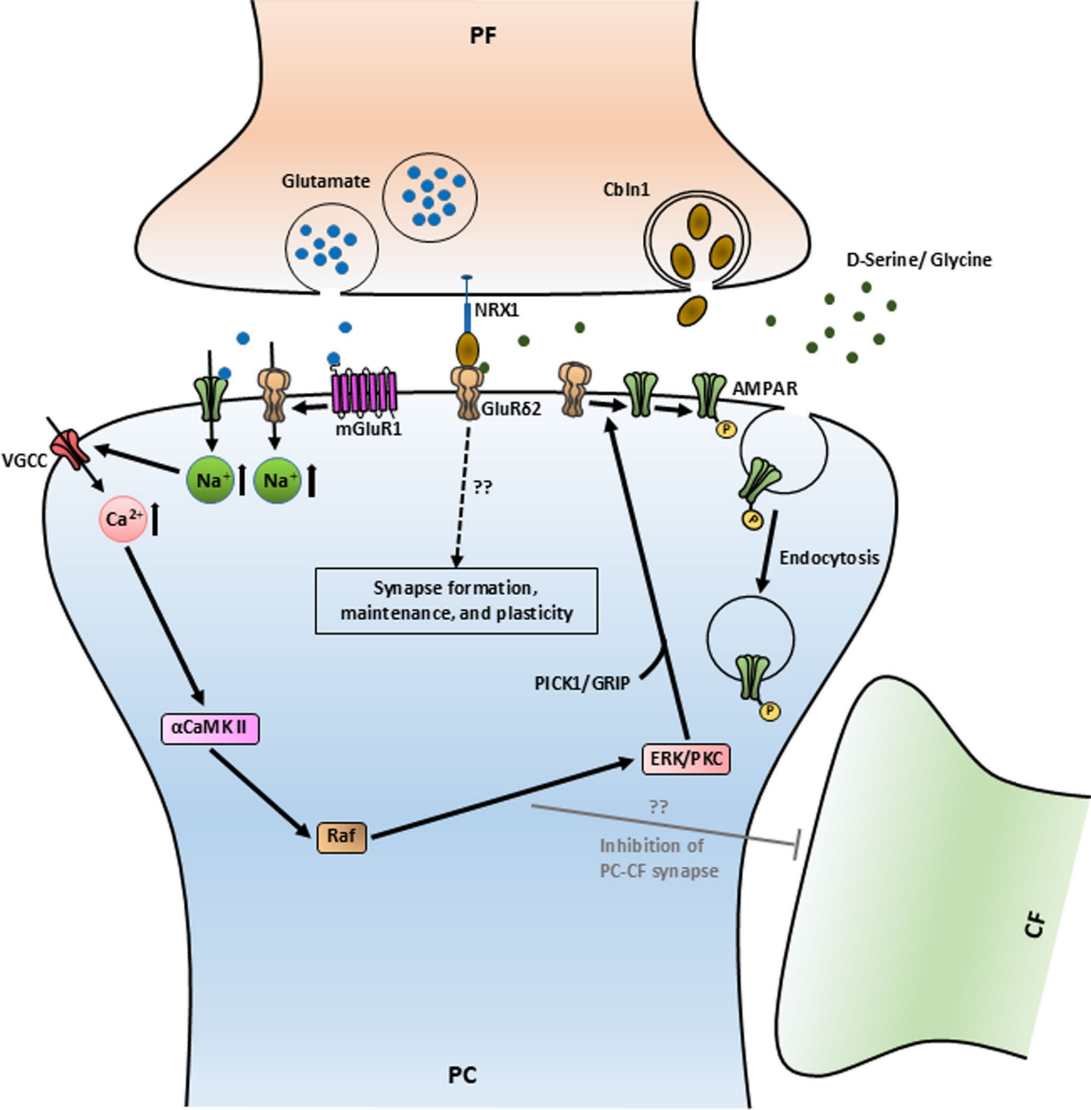
Molecular cascade illustrating the involvement of GluD2 or GluRδ2 in synaptic plasticity, development and maintenance of PF-PC synapses. Black arrows indicate pathways involved in mGluR1 mediated LTD induction through GluRδ2/PKC pathway. The grey arrow represents the direct or indirect inhibition of PC-CF synapse by mGluR1-mediated PKC/ERK pathway. The dotted arrow represents the involvement of the tripartile complex in synapse formation, maintenance, and plasticity.?? - mechanism that remain unclear

GluRδ2 is mainly present in the synapses between the granular cell (GCs) parallel fibers (PFs) and PCs [23]. The main roles of GluRδ2 are development and plasticity of the PF-PC synapses and the synapses between climbing fibers (CFs) and PCs (Fig. 1).

### Role of GluRδ2 in Synaptic Plasticity

Long-term depression (LTD) in the cerebellum is a type of synaptic plasticity and one of the most critical mechanisms for motor learning [24–27]. LTD in PCs is initiated with the release of glutamate in the PF-PC synapses by the PFs [28, 29]. Glutamate activates the GluRδ2 by triggering the glutamate-induced metabolic glutamate receptor type 1 (mGlu1) [30], which leads to Na^+^ influx. The increase of intracellular Na^+^ leads to a rise in intracellular Ca^2+^ via the voltage-gated calcium channel (VGCC) and ultimately resulting in the activation of α calmodulin-dependent protein kinase II (αCaMKII) [31–34]. αCaMKII activates Raf by phosphorylating it, which leads to the LTD by the protein kinase C /extracellular signal-regulated kinase (PKC/ERK) pathway [34–36]. Protein interacting with C kinase 1 (PICK1), which binds to the GluRδ2 via the PDZ domain [37], provides binding domains for PKC and glutamate receptor-interacting proteins (GRIP), which ultimately helps in phosphorylation and the internalization of AMPAR [37–39]. The reduction of AMPAR decreases the response (depolarization) of PCs to glutamatergic signaling and thereby depresses their firing activity, i.e., induces LTD. GluRδ2-regulated LTD is essential for fine-tuning the strength of PF-PC synapses and thereby for motor learning. Experimental evidence was provided by the disruption of GluRδ2 or αCaMKII, which both led to deficits in motor learning [24–26, 33].

### Role of GluRδ2 in Development and Maintenance of PF-PC Synapses

Various studies showed that LTD takes place in both PF-PC synapses and inferior olive’s CF-PC synapses [40–44]. Generally, CFs make synapses with the primary dendritic branches of PCs and PFs form synapses with the secondary and tertiary dendritic branches of PCs [43, 45]. During development, multiple CFs innervate one immature PC, but gradually the CF-PF synapse is eliminated, and one CF forms a synapse with one PC (mono-innervation) by approximately postnatal day 20 in mice [25, 46–48]. In immature PF-PC synapses during postnatal days 1 and 7, GluRδ2 is distributed among the dendritic shafts and spines. Although, on postnatal day 7, synaptic contact between PCs and PFs has been seen, indicating the ability of newly formed PCs to create synapses with PFs without much influence of GluRδ2 [49]. However, from postnatal days 10 to 14, dendrites of PCs grow extensively and the presence of GluRδ2 becomes exclusively abundant at their dendritic spines, leading to synaptic targeting [16, 49]. In GluRδ2 mutant mice, leaky GluRδ2 causes a disturbance in the signal transduction pathway including mGluR1/PKC, which is shown to be a crucial factor in CF synapse elimination [16, 50–53]. Due to this leaky receptor, multiple CFs form synapses, not only to the primary dendrites but also to secondary and tertiary dendrites of one PC [54–57]. Expression of GluRδ2 induced by the Sindbis virus injected into the subarachnoid space partially recovers PF-PC synapse formation, and alleviates motor dysfunction in GluRδ2 null mice [25, 58]. In addition, more PFs form synapses when GluRδ2 expression is induced by tetrodotoxin [55, 59]. Thus, the GluRδ2 receptor favors PF-PC synapses over CF-PC synapses, which helps in the formation and maintenance of PF-PC synapses [26, 46].

Besides glutamate-induced LTD, *g*lycine and D-serine also reduce currents through GluRδ2 [20, 21]. The proper mechanism of this glycine- and D-serine-induced LTD is still unclear. Glycine and D-serine binding is facilitated by the presence of cell-to-cell contact with cerebellin-1 (Cbln1) and the Neurexin-1β(NRX1) dimer in the N-terminal domain of the GluRδ2 receptor [60]. Cbln1 and NRX1 are predominantly expressed by PF of the GC [61, 62]. Cbln1 null mice show similar cellular and physiological abnormalities as mice lacking functional GluRδ2, including Lurcher mice [61]. Administration of recombinant Cbln1 induced new PF-PC synapses in vitro, transiently restoring PF-PC synapses in vivo, and completely rescued severe ataxia in Cbln1 mutant mice [63]. Therefore, Cbln1-NRX1-GluRδ2 tripartite complex has also been suggested to promote PF-PC cell synapse formation and maintenance [64–66].

## Lurcher Mutation and Neuropathology

### Morphological Change in the Lurcher Cerebellum and Inferior Olive

**Fig. 2:**
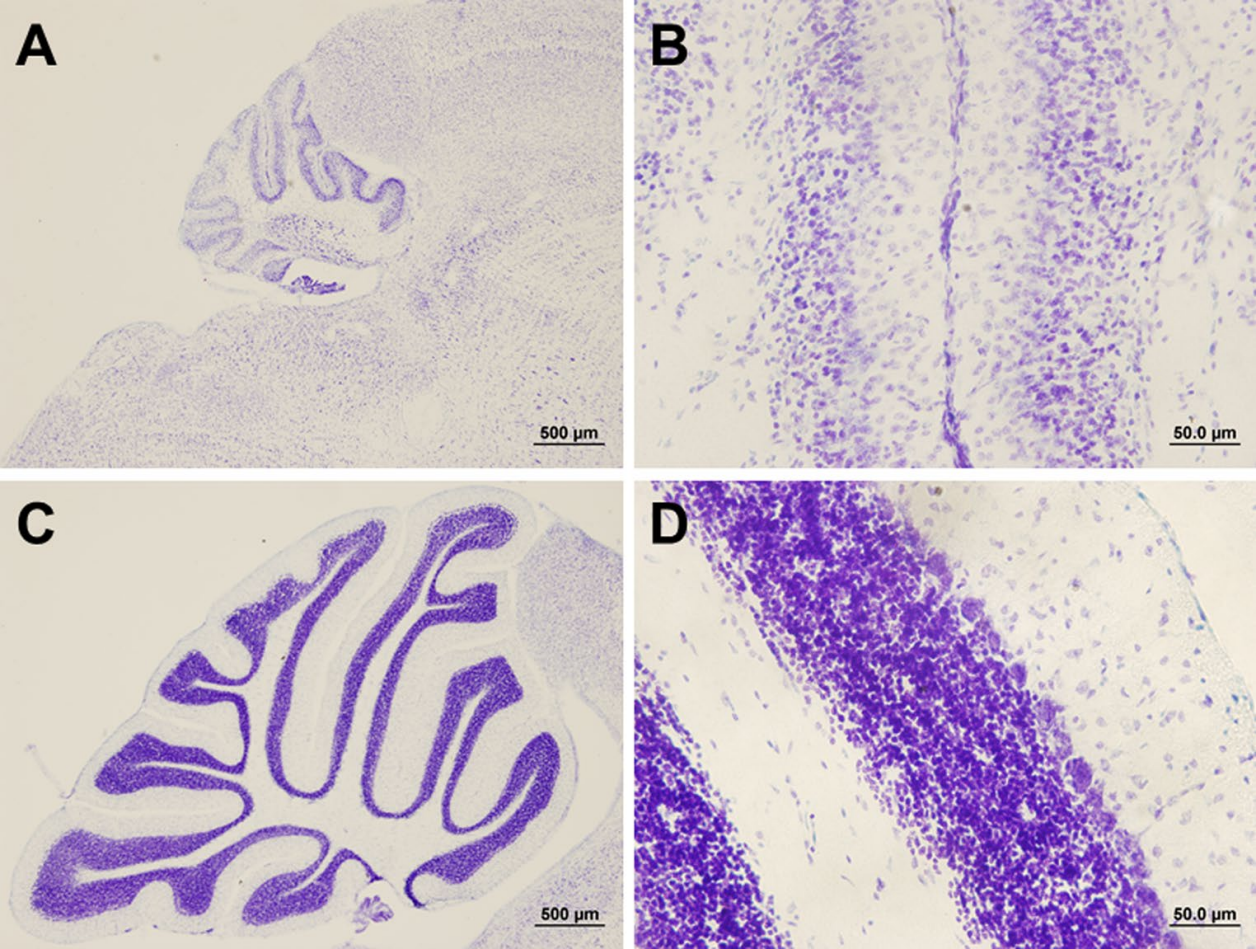
The cerebellum of a Lurcher mouse (**A**, **B**) and a wild-type mouse (**C**, **D**). Sagittal sections, Nissl staining with cresylic violet

**Fig. 3:**
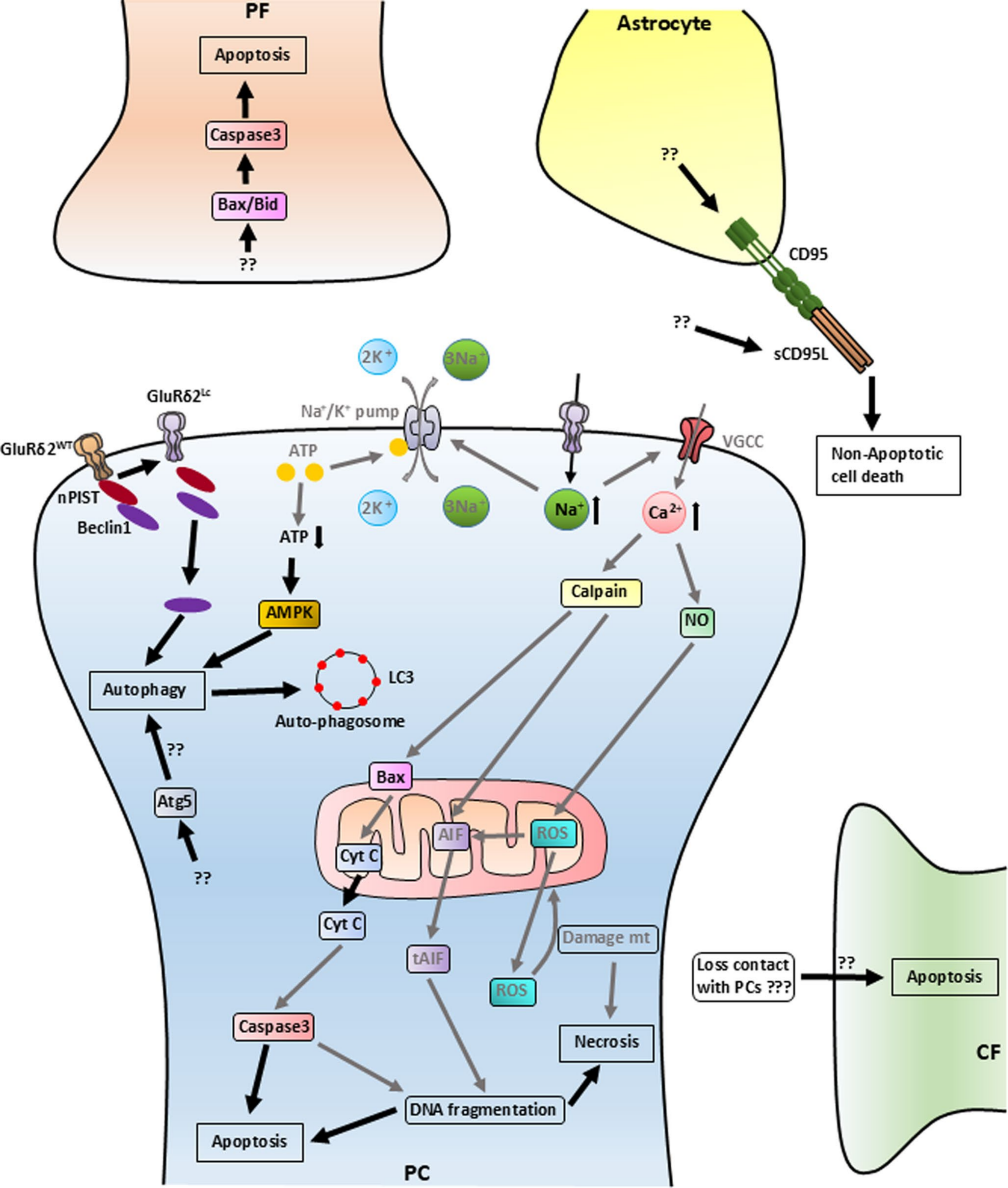
Molecular cascade of the involvement of GluD2 or GluRδ2 in cerebellar cell death. Black arrows and texts represent pathways/molecules, respectively, proved to be directly involved in degeneration in Lurcher mice. Gray arrows and texts are used for pathways/molecules, respectively, involved in cell death mechanisms in general, but not being proved to be specifically enhanced in Lurcher mice. Because of their relation to components of the cascades that are modified in Lurchers, their direct or indirect role can be hypothesized.??– unknown signals and components of the signaling pathway.???– Hypothesis presented by many authors [70, 72, 76–78]

Compared to non-mutants (called wild-type mice, WT), the cerebellum is reduced to approximately 71% in Lurcher mice, where 95% of the PCs die between postnatal days 8 and 25 [67, 68] (Fig. 2). The death of granule cells (GC), other cerebellar cortex interneurons, and inferior olivary (IO) neurons is induced by a secondary degradation process between postnatal days 8 and 11 [67–70]. Various studies revealed that PCs in Lurcher mice die via different cell death mechanisms– necrosis, apoptosis and autophagy [71–75] (Fig. 3). The presence of active apoptotic protein caspase 3 has been found in all these cell types, PCs, GCs and IO neurons [72]. Blocking the Bax apoptotic pathway, in which caspase 3 is a downstream protein, leads to the survival of GCs, but not to the survival of PCs and IO neurons in Lurcher mice [72]. These data suggest that the Bax apoptotic pathway is exclusively responsible for the death of GCs, but not for the death of PCs and IO neurons.

### Primary Effect of Lurcher Mutation and Cell Death Mechanisms

There are two different Lurcher mutations, *Grid2*^*Lc*^ and *Grid2*^*Lc − J*^, both being base pair point mutations (G-to-A substitution) between markers D6Rck353-D6Rck357 and D6Rck314-D6Rck361 respectively, changing a non-polar alanine to polar threonine (A654T) in the transmembrane domain III, leading to the leaky GluRδ2 and both causing similar phenotype [16, 17, 79]. Leaky GluRδ2 in PCs of Lurcher mice leads to increased intracellular Na^+^ [80], which activates the voltage-gated calcium channels and the increased influx of Ca^2+^ [31]. Increased intracellular Ca^2+^ also promotes the production of nitric oxide (NO) and reactive oxygen species (ROS) through mitochondrial stress, which leads to necrosis [81, 82]. Increased intracellular Ca^2+^ activates a calcium-dependent cysteine protease, Calpain [83]. Calpain promotes activation of the mitochondrial-bound apoptosis-inducing factor (AIF) to truncated AIF (tAIF), which modulates DNA fragmentation leading to apoptosis and necrosis [84–87]. Calpain also activates the intrinsic apoptotic pathway by activating the Bcl-2 pathway (Bax/mitochondrial CytC) and activating caspase-3, which also leads to DNA fragmentation [84, 88–90]. Translocation of CytC from the inner mitochondrial membrane into the cytosol is also seen in Lurcher mice [91], which proves Calpain-mediated cell death via CytC in Lurcher mice. Due to increased Na^+^ inside the cell, the Na^+^/K^+^ pump uses more ATP to normalize the cell from Na^+^ ion overdose, which is a result of a leaky GluRδ2 channel. High ATP consumption leads to a lack of ATP inside the cell, which activates the AMPK pathway of autophagy, which is seen to be higher in Lurcher mice [92, 93]. In the healthy state, the C terminal of GluRδ2 provides a binding site for the PDZ domain of a trans-golgi associated protein known as nPIST [75], which has an important role in vesicular trafficking [94]. This complex further interacts via the coil domain of nPIST with the coiled domain of Beclin1 [75], a protein known for its role in the autophagy pathway [95]. It has been reported that Beclin1 also interacts with Bcl-2 [96]. Release of this complex from the mutated GluRδ2 leads to the autophagy pathway, as Yue et al. have found that the absence of the PDZ domain resulted in Beclin1-induced autophagy [75]. Studies have found that there is an increase of phosphorylated MAP1B bound LC3 in Lurcher mice, which leads to the presence of high numbers of LC3-labeled autophagosomes in the axon terminals of PCs [81, 97]. The presence of autophagosome-like vacuoles in the dendrites, soma, and axons of PCs is well documented, although the presence of autophagosomes is far more than in the dendrites and soma [75, 97]. It has been shown that atg5 (an autosomal marker) null mice crossed with Lurcher mice have an increase of PCs’ death, more than Lurcher mice with atg5, which eventually suggests that atg5 rather protects the PCs in Lurcher mice than causes cell death by autophagy [98].

Many studies speculated that the loss of contact with PCs is one of the reasons for the death of IO neurons and other neurons including GCs, basket cells, and stellate cells [70, 72, 76–78]. However, the precise mechanisms involved in the degradation of CFs and IO neurons remain to be expounded. The increase of GFAP indicates the activation of astrocytes [99], and CD95 is known to have roles in both inflammatory and apoptosis processes [100–102]. In Lurcher mice, the elevation of CD95 and GFAP has been seen in the cerebellum [103, 104]. More particularly, Vernet-der Garabedian et al. found that neuronal death triggered a glial reaction, in which the CD95/CD95L system is involved and acts through the non-apoptotic pathway in the cerebellum of Lurcher mice [104].

### Changes in Signaling Molecules in Lurcher Mice

Neurodegeneration in Lurcher mice is accompanied by changes in several signaling molecules (Table 1). PCs are GABAergic projection neurons by nature [108]. As a result of the loss of PCs in Lurcher mice, there should be lower inhibitory postsynaptic currents (IPSC) in the brain. However, studies have shown that there are compensatory mechanisms for the loss of PCs by the increase in the number and size of other interneuron GABAergic synapses, due to which there are no differences in the IPSCs between Lurcher mice and WT mice [109, 110]. On the other hand, in the Lurcher cerebellum, a decrease in glutamate neurotransmitter concentration has been observed [105], which is mainly released by the granular cells in the cerebellum [111]. Additionally, an increased serotonin concentration and reorganization of serotonergic innervation in the cerebellum of Lurcher mice have also been reported [106].

**Table 1:** Concentration of different neurotransmitters in the cerebellum and other brain parts in adult Lurcher mice, compared to WT mice

Neurotransmitter	Cerebellum	Other brain part	References
GABA	↔	↓ entorhinal cortex	[105]
		↔ hippocampus	
Glutamate	↓	↓ entorhinal cortex	[105]
		↓ hippocampus	
Glycine	↔	↓ entorhinal cortex	[105]
		↔ hippocampus	
Serotonin	↑	↔ whole brain	[106]
Dopamine	**	↔ whole brain	[105, 107]

↓ = lower concentration; ↑ = higher concentration; ↔ = no change in concentration, ** = data unavailable

The changes in the neurotransmitter concentration associated with cerebellar atrophy in Lurcher mice, resulting from the mutation in GluRδ2, are not exclusive to the cerebellum, but also affect other regions of the brain (Table 1). Thus, there is a decrease of GABA, glycine, and glutamate neurotransmitters in the entorhinal-piriform cortex, and a change of glutamate concentration, but not of GABA and glycine, in the Lurcher mouse hippocampus [105]. Although the cerebellum has projection to the dopaminergic neurons in the midbrain [112, 113], there is no change of dopamine in the brain, which might be due to the mild degradation of the cerebellar nuclei (33% [107]) or due to the parallel degradation of both cortex and nuclei, so that both areas can not affect the dopaminergic neurons in the midbrain [105].

In the Lurcher mutant cerebellum, also inconstant changes in trophic factor levels [103, 114, 115], significant changes in the vascular bed [68], changes in inflammatory cytokines [116] and immune cell and organ abnormalities [117, 118] have been found. Such biochemical changes are direct or indirect effects of the mutation and primary cell loss, but could potentially contribute to the development of secondary neuropathologies and functional changes in the brain of Lurcher mice.

## Lurcher Phenotype

### Motor Syndrome

The name Lurcher came from the ataxic lurching gait of the mouse, which is easily recognizable. Philips (1960), who first described Lurcher mice, reported that they usually tend to walk backwards [15]. In 1987, Rossignol’s group made a thorough analysis of the locomotor problems of Lurcher mice. They described that Lurcher mice show sharp and irregular vertical hip movements, which indicates that they are not able to properly produce the sequence of limb movements necessary for smooth walking. Electromyography (EMG) revealed differences in the activity of the *tibialis anterior* and *triceps surae* muscles in the hindlimb of Lurcher mice, compared to WT mice [119]. There are also overlaps in the activity of the extensor muscle of the hindlimb and the flexor muscle of the forelimb in Lurcher mice [119]. Some of the gait parameters, however, depend on the walking speed of the mice, which is much slower in Lurcher mice than WT mice [120].

The number and duration of several grooming movements are also reduced in Lurcher mice [121]. This has mainly been explained by poor maintenance of body balance, when some of the extremities are used for grooming instead of supporting the body [121]. On the other hand, the body shaking behavior that allows body support by all four limbs is not disturbed in Lurchers [121]. Therefore, changes in grooming are supposed to be rather motor and balance problems and not due to behavioral abnormalities or hypoactivity [121]. Hypoactivity is not present in Lurcher mice, since they have similar activity levels in the T-maze and open field compared to WT mice [121–123].

Lurcher mice show structural changes in the skeletal muscles and tibial cortex and also in the myocardium [124, 125]. Skeletal muscle and bone changes can most probably be explained by the abnormal movements of ataxic mice [124, 125]. As there is little evidence of the expression of GluRδ2 receptors in the myocardium, we can speculate about the effects of vegetative and hormonal regulations related to higher stress reactivity and abnormal motor activity in Lurchers [126].

Lurcher mice are inferior to WT mice in many specific motor tasks (Table 2). In the well-known rotarod test to investigate motor equilibrium and coordination in rodents [136], both adult and juvenile Lurcher mice fell sooner than WT mice [137, 139]. They also exhibit poor performance in the tilted platform test, which measures the maintenance of body equilibrium [146, 155, 156]. In addition, both age groups of Lurcher mice performed worse than WT mice in the treadmill test [138], an assay for motor coordination and muscle performance [140, 141]. In tests on the principle of suspension on a horizontal bar (such as suspended wire, coat hanger, or vertical grid), which requires both muscle strength and balance, both adult and juvenile Lurcher mice fell more frequently or sooner than WT mice [137, 139, 151–154].

**Table 2:** Parallelization of specific motor components affected between human patients with cerebellar motor syndrome and Lurcher mice

Cerebellar motor syndrome			Lurcher mice		
References	Detection method (test)	Similar/analogous symptoms		Detection method (test)	References
[127–130]	Clinical Gait Assessment, SARA and BARS2 assessments by inertial sensors	Impaired gait parameters (bipedal)	Impaired gait parameters (quadrupedal)	Automated gait analysis systems based on the overground principle	[114, 120]
[129, 131, 132]	Electromyography	Irregular muscle activities	Irregular muscle activities	Electromyography	[119]
[127, 133–135]	Appendicular examination, Heel–shin test	Impaired motor coordination	Impaired motor coordination	Rotarod test, Treadmill test	[136–141]
[142–145]	Romberg test, SARA balance score, Berg Balance Scale (BBS) score	Impaired static equilibrium	Impaired static equilibrium	Assessing grooming behavior, Unsteady platform test	[121, 146]
[142, 147, 148]	Sensory Organization Test (SOT) and Limits of Stability (LOS), Berg Balance Scale (BBS) score	Impaired dynamic equilibrium	Impaired dynamic equilibrium	Rotarod test, Wooden beam test (in adults)	[136, 137, 139]
[135, 149, 150]	Patellar reflex test, Hydraulic hand dynamometer test, Pronator drift test	Impaired muscle strength/ performance	Impaired muscle strength/ performance	Treadmill test, Horizontal bar test	[137–141, 151–154]

Interestingly, while both the distance traveled and latency before falling are lower in adult Lurcher mice, there are no significant differences for juvenile Lurcher mice on the rectangular stationary beam, which provides more grip than the round stationary beam, where both adult and juvenile mice fell sooner than WT mice [137, 152, 153].

Although Lurcher mice suffer from severe motor disorders, they show motor learning ability– they improved their performance upon repeated trials, especially in the rotarod test [137, 157, 158] and in the tilted platform test [146, 156]. In the rotarod test, Lurcher mice adopt a walking behavior, which improves their performance [137]. Although Lurcher mice do not have any significant difference in latency before falling in the wooden beam test, they have been seen to spend more time walking than WT mice over repeated trials, which is most likely a strategy to regulate and maintain body equilibrium to prevent them from falling [119, 137]. It has been shown that cerebellar nuclei are involved in the elaboration and execution of anticipatory body adjustments, as motor learning is absent in Lurcher mice after cerebellectomy that also removes the nuclei, which otherwise show only moderate degeneration [107, 159, 160]. However, the ability of motor learning in Lurcher mice decreases with disease progress and aging [137]. This can be justified by studies showing high metabolic activity in the cerebellar nuclei in Lurcher mice, which could lead to high oxygen free radical and neurons’ death due to toxicity in older Lurcher mice [137, 154].

### Cognitive Deficits

The involvement of the cerebellum in cognitive functions was proven by multiple studies in both humans and animals (for review, see [6, 161, 162]). The anatomical connections of the cerebellum to different brain areas involved in cognitive processes are also well documented and support the hypothesis on the multiple roles of the cerebellum (for review, see [163, 164]). Accordingly, Lurcher mice have specifically impaired cognitive abilities compared to WT mice (Table 3).

**Table 3:** Comparison of signs of CCAS in human patients and cognitive and behavioral abnormalities in Lurcher mice

CCAS patients			Lurcher mice		
References	Detection method (test)	Similar/analogous symptoms		Detection method (test)	References
[9]	Short- and long-term memory tests: word immediate recall, words delayed recall, verbal paired associates	Impaired memory (in general)	Impaired memory (in general)	Left/right discrimination in T- maze, radial maze, Z-maze, Object localization task, MWM	[165–172]
[6, 9]	Forward digit span, reverse digit span, months backward task	Impaired working memory	Impaired working memory	Object localization task, Left/right discrimination in T- maze	[168, 171, 173]
[9]	Verbal associative learning	Impaired associative learning	Impaired associative learning	Classical conditioning task	[170]
[6, 9, 10, 174]	Cube/pentagon test, judgement of line orientation	Visual-Spatial impairment	Visual-Spatial impairment	MWM	[165–170]
[9]	Letter-number sequencing, category switching, trails A and B task	Mental inflexibility	Behavior inflexibility	Reverse conditional visual discrimination task, changing platform position in MWM*	[175]
[9]	Go/no-go task	Behavioral disinhibition	Behavioral disinhibition	Elevated plus maze, predator proximity test, prepulse inhibition examination	[155, 170, 176, 177]
[12, 178]	SCID** scores, psychiatric interviews	Depression and anxiety	High stress response and anxiety	Corticosterone measurement	[155]
[6]	Case study, clinical observations	Attention deficits, hyperactivity	Increase spontaneous activity	Open field, hole- board test	[177, 179, 180]

*MWM = Morris Water Maze

**SCID = Structured Clinical Interview for DSM (Diagnostic and Statistical Manual for Mental Disorders)

#### Spatial Impairments in Lurcher Mice

Although the essential structure for spatial memory and orientation is the hippocampus, deterioration of spatial abilities is also observed in cerebellar patients with CCAS, as well as in animal models of cerebellar diseases [6, 9]. The mutation in PP2B and PKC1 protein, involved in the neuronal plasticity in the cerebellum, leads to altered place cell activity or an unstable cognitive map in the hippocampus [181, 182]. Therefore, it is not surprising that Lurcher mice, as well as many other cerebellar mutants, fail in spatial tasks (for review, see [166, 183]).

In the object localization task, where identical objects were presented for 3 days in a certain spatial configuration and changed to a different configuration on the 4th day, Lurcher mice had fewer sniffing responses than WT mice, suggesting that Lurchers were less able to recognize the change and were less interested in a novelty situation than WT mice [171]. As a dim red bulb illuminated the experimental room and the effect of visual cues could be excluded, Belzung et al. suggested that the behavior of Lurcher mice could be considered as improper cognitive mapping of the space, or impaired spatial memory [171].

In the Z-maze filled with water, where mice have to find an escape platform, Lurchers also exhibited impaired spatial learning, committing more errors, and had longer escape latencies compared to WT mice [172]. Conversely, mutants displayed different behaviors, depending on the protocol’s paradigm during T-maze left-right discrimination. Lurcher mice showed no difference from WT mice in left-right discrimination in the dry T-maze with a food reward [168]. When the T-Maze was filled with water and the escape platform was visible, they displayed impaired performance [168, 171]. It should be noted that the water arrangement of the T-maze probably represents a more stressful condition that might influence the behavior of mice during the task (see below). Surprisingly, if the platform was hidden, Lurcher mice achieved a better score of correct arm entries, which Belzung et al. explained as a result of the win-stay strategy [171], which is achieving success by repeating an earlier successful action that leads to a positive outcome [184]. This suggests that the selection of a simpler strategy could be an efficient adaptation to a cognitive disability.

Furthermore, Belzung et al. reported that in the radial maze test, where the first entry but not the repeated entries to each arm were rewarded by food, Lurcher mice made more repetitive arm entries, counted as incorrect responses, than WT mice [171]. They proposed that mutants followed the simple win-stay strategy in the radial maze, as in the T-maze [171]. In contrast, WT mice, exploring different arms, adopted a win-shift strategy more efficiently for this task. The impaired behavior of Lurcher mice in the radial maze also can be considered as impairment in a reference memory task which needs precise cognitive mapping [171].

One of the widely used behavioral paradigms for rodents to check visuospatial memory is the Morris water maze (MWM), where the animals need to find an escape platform in a pool filled with water, allowing different arrangements with distal or proximal visual cues and with a hidden (spatial learning task) or visible (visual guidance task) platform. Both young and adult Lurcher mice had difficulties in both the visual guidance and the spatial learning tasks in the MWM. Although Lurcher mice were able to find the platform often without any external help and to learn, as demonstrated by escape latency shortening [180], their escape latency was always longer than that of WT mice in both the visible and hidden platform tasks in the MWM [165–170]. Additionally, besides a specific learning deficit or impairment in visual-spatial abilities, many potential factors can explain the inferior performance of Lurcher mice in the MWM and other spatial learning tests.

For instance, learning is highly dependent on motivation, also in tests developed for laboratory rodents [180, 185–187]. Motivation is related to emotions as indicators of the biological importance of the stimuli. The cerebellum is believed to be involved in emotional processes, and cerebellar patients display emotional changes [12, 178].

Fortier et al. argued that Lurcher mice have no difficulties in swimming, as their swimming speed is similar to that of WT mice [119], concluding that the impairment in the water T-maze and MWM tests are not associated with an effect of motor disturbances. On the other hand, although Lurcher mice have similar swimming abilities, they deviate more in the swimming path and have inferior heading error parameters than WT mice, suggesting that the inability to maintain a straight direction might contribute to inferior performance in water maze tasks [180, 186, 188]. Alternatively, failures in spatial learning and orientation tests might also be explained by deficits in visuomotor coordination [189, 190].

In addition, water exposure-induced stress could influence performances in water mazes [191]. It has been found that in Lurcher mice, the corticosterone level increases more than in WT during water exposure [186]. Since Lurcher mice have permanently higher stress reactivity, their hippocampus can be affected by corticosterone and reduce its learning capacity [192, 193]. Exposure to water causing acute stress at the moment of the test might induce behavioral disinhibition (see below) and maladaptive behavior that worsens performance in the test [155, 170]. Higher anxiety can increase thigmotaxis (preference of the periphery of the maze), which is in fact a maladaptive strategy, reducing the chance to reach the platform which is at some distance from the pool periphery. On the other hand, a longer distance moved during a certain period in the MWM, due to a higher average swimming speed or the absence of immobility responses (one of the potential outcomes of disinhibition), can reduce escape latency in Lurcher mice and increase the chance of randomly locating the platform [180, 186, 194].

Nevertheless, stress response does not seem to be the only or the main factor, since Lurcher mice showing improvement in the MWM task did not reduce stress response levels as measured by corticosterone levels [186]. Taken together, all these data and considerations suggest that Lurchers’ poor performance in both visible and hidden platform tasks in the MWM is of a complex origin, with the potential contribution of multiple mechanisms.

In conclusion, the poor performance of Lurcher mice in tests of spatial learning and orientation cannot be attributed only to impairment of these specific functions, but there can be a substantial contribution of other pathological phenotypic traits, more or less directly related to cerebellar dysfunction. Similar problems in interpretation can also occur in human cerebellar patients. Patients with ataxia with oculomotor apraxia, classified under oculomotor apraxia-associated autosomal recessive cerebellar ataxia, show impairment in cognitive tests. However, in those patients, performance could be negatively influenced by malfunction of the oculomotor components caused by apraxia impairing their visual inputs [195, 196].

#### Classical Conditioning Changes in Lurcher Mice

Lurcher mice also show changes in classical conditioning, interpretation of which appears to be easier compared to the assessment of more complex spatial tasks. In the eyelid conditioning test, Lurcher mice showed a similar learning curve in the acquisition of eyelid response to WT mice, although the amplitude of the response was much lower than in the WT individuals [170]. From these results, it seems that the cerebellar cortex is important for the quality and quantity of the generated conditioned response, but not for the acquisition of conditioned responses. Later, it was found that in Lurcher mice, the absence of a functioning olivocerebellar system, important for maintaining information and general dynamic control during learning tasks [197–199], is compensated for by the collaborative action of the interpositus (IPN) and red nuclei. This compensation helps in maintaining the acquisition of eyelid responses similar to WT mice [200]. Other research has found that proper IPN firing is important in the eyeblink responses in conditioned response (CR) and unconditioned response (UR) for both delay and trace paradigms [201]. The change in the firing rate of IPN can also explain the impaired performance in the eyeblink response of Lurcher mice compared to WT mice [201]. From these studies, it can be concluded that, due to olivocerebellar degeneration, Lurcher mice have impaired performance in CR and UR, but not in the acquisition of conditioned eyelid responses, due to compensatory mechanisms.

#### Cognitive and Behavioral Inflexibility in Lurcher Mice

In the conditional visual discrimination task, where the condition of getting a reward were reversed after acquisition trials, Lurcher mice adjusted their behavior according to the reversed task, but still made more errors than WT mice [175]. This result suggests that they had learned about the changes in the task, but were not able to adapt to the new reversed task, indicating behavior inflexibility [175], which is also seen in CCAS human patients [9]. Using chimeric Lurcher mice in this task helped to estimate the effect of PCs’ degradation on cognitive processing, as the chimeric Lurcher mice, with some healthy PCs, made fewer errors than Lurcher mice with no Purkinje cells and more errors than WT mice in the reversed task. Similarly, after changing the platform position in the MWM, Lurcher mice had difficulty in locating the platform and learning its new position, preferring to search for the platform in the original quadrant instead [180], also suggesting reduced cognitive or behavioral flexibility.

### Emotional Reactivity and Behavioral Changes

In line with cerebellar involvement in behavioral control and behavioral and emotional components of the CCAS in human patients [5, 6, 9–12], cerebellar mutant mice including Lurchers also show a variety of pathological behavioral traits.

#### Anxiety, Behavioral Disinhibition and Stress

Lurcher mice show abnormal behavior in anxiogenic conditions (Table 3). For example, in the acoustic startle response and prepulse inhibition test, Lurcher mice show a similar startle response to WT mice, but are impaired in producing prepulse inhibition [170]. Reduced prepulse inhibition in Lurcher mice might be due to the behavioral disinhibition induced by stress, or due to deficits in sensory integration resulting from olivocerebellar degeneration [155, 170]. Additionally, the behavior of mutants diverged extremely from that of the controls in the elevated plus maze, which is used as a test of anxiety. They spend more time in the anxiogenic open arms of the maze [155, 177]. Furthermore, mutants spent more time in the proximity of a rat, a natural predator of mice, in an ethological test of fear [176]. They also showed low immobility responses and more dispersed activity than WT mice in the forced swimming test, which indicates their lack of learned helplessness due to stress triggered by water [180, 202]. These apparently less anxious behavioral features could also be explained by stress-induced behavioral disinhibition [155, 170], rather than by lower anxiety, i.e., Lurcher mice have a reduced ability to inhibit exploration of a novel aversive environment (elevated plus maze) or novel object (rat). This reduced avoidance of aversive stimuli was considered as anxiety-related behaviors in these mutants. In fact, the presence of anxiety, potentially even strong anxiety, is suggested by the higher elevation of corticosterone, an indicator of stress response upon exposure to these anxiogenic conditions– elevated plus maze, water, rat [155, 203]. However, Lurcher mice exhibit less exploratory behavior, although with an increase of spontaneous activity in the hole-board test [177, 179] and open field test [180]. This contrasts with the results from the elevated plus maze and predator proximity tests [155, 176, 177]. It could be due to the lower anxiogenic potential of the hole-board and open field arena, compared to the elevated plus maze or even a rat presence. Another phenomenon that could be hypothetically attributed to behavioral disinhibition is the high incidence of maternal infanticide in Lurcher mouse dams [204].

A stronger increase of corticosterone was found in Lurcher mice also upon the MWM test along with having larger adrenal glands compared to WT mice, indicating a high-stress condition with the abovementioned impacts on behavior [186]. Of course, the corticosterone level shows the intensity of adrenal cortex activation and not directly how anxious the mouse is feeling. On the other hand, using metyrapone, a CORT synthesis inhibitor, reduced blood corticosterone levels in Lurcher mice, but did not affect their behavioral disinhibition [205]. This suggests that hyperreactivity of the stress response is not just at the peripheral level of corticosterone synthesis, but at least the behavioral reaction to stress is mediated on a more central level without a need for the corticosterone effect. Interestingly, when Lurcher mice are allowed to become familiarized with the experimental arena, their behavior improves [206]. This familiarization effect is also reflected in their blood corticosterone levels, which decrease once the mice become more accustomed to the arena before behavioral testing [206].

#### Unknown Mechanisms of Behavioral Changes

The precise identification of the impaired neuronal network and the molecular mechanisms underlying behavioral and emotional changes, particularly behavioral disinhibition in Lurcher mice, are still unknown. One of the important factors could be the tonic inhibition that the cerebellum exerts on the amygdala, hippocampus, and septum involved in the Papez circuitry [113] and that might be reduced by cerebellar degeneration in Lurcher mice. Behavior is also influenced by learning capacity, which is reduced in Lurcher mice (see above). Therefore, inability to learn can lead to repetition of previously ineffective solutions, inability to adapt to new conditions, reduced efficiency of activities and in corollary to dissatisfaction, depression and anxiety.

## Comparison of Cerebellar Syndromes in Humans and Lurcher Mice

In 2019, in their article concerning the model validity for preclinical studies, Tadenev and Burgess mentioned that “It is important to remember that all models are models, and their limitations must be considered, as well as their potential.” [207]. In the following section, we propose to analyze the potential validity of Lurcher mutant mice as a model for cerebellar syndromes, regarding the three classic types of validity: construct, face, and predictive.

### Construct Validity

The ***construct validity*** of disease models is determined by whether the etiology and pathogenesis leading to the disorder in the model correspond to those of the human disease. On a general level, in both Lurcher mice and human cerebellar patients, cerebellar dysfunction leads to behavioral and cognitive alterations (in humans defined as CCAS) and cerebellar motor syndrome (particularly cerebellar ataxia, which is well visible in both humans and cerebellar mutant mice, the mechanisms of which are relatively well understood). More specifically, it is important whether the cerebellar motor syndrome and cognitive affective syndrome (syndromes are complexes of symptoms that may have diverse etiologies, i.e., are not diseases *sui generis*) result from the same neuropathologies in the mouse model and human patients, including potential secondary brain changes and the pattern of neuropathological development and whether there are analogous mechanisms of links between the neuropathologies and functional impairments (unfortunately not completely known particularly for the CCAS). If considering Lurcher mice as a model of a particular disease (nosological unit, disease *sui generis*), identical etiology and primary mechanisms of its action on the organism are also of importance.

This review has already discussed the loss of Purkinje cells and other secondary structures in Lurcher mice. Juxtaposed with the Lurcher mice, where degradation mainly starts due to the mutated GluD2 receptor, CCAS patients have a variety of cerebellar disorder etiologies. In 1998, Schmahmann and Sherman conducted a study on 20 etiologically different patients with cerebellar disease, which included patients with stroke, postinfectious cerebellitis, cerebellar cortical atrophies, and midline cerebellar tumor resected [5, 6]. Recent studies also confirmed other cerebellar pathological conditions to be related to CCAS, such as posterior fossa injury, spinocerebellar ataxias types 5, 6, and 8 (SCA5, SCA6, SCA8), autosomal recessive cerebellar ataxia type 1 (ARCA1), episodic ataxia type 2 (EA-2), and idiopathic late-onset cerebellar ataxia (ILOCA), which are isolated cerebellar pathologies [9]. CCAS can also be seen in complex cerebrocerebellar pathological conditions, such as cerebellar and brainstem hemorrhage, complex cerebrocerebellar degeneration with gene variants (senataxin, gene, and X-linked recessive *ATP2B3* gene), pontine cavernous malformation, ataxia with oculomotor apraxia type 2 (AOA2), Friedreich’s ataxia, SCA1, SCA2, SCA3, SCA7, SCA17, and multiple system atrophy of the cerebellar type (MSA-C) [9].

These pathologies arise acutely or develop slowly as progressive degenerations during a patient’s lifetime. Some of them affect children (degenerations with early onset, such as Friedreich’s ataxia, injuries, and tumors in children). In Lurcher mice, cerebellar degeneration is a specific disease having a particular etiology (Lurcher mutation– see above), well-determined and constant olivocerebellar neuropathology, and a very early onset and rapid progress that can determine secondary adaptations or maladaptations of the developing brain via mechanisms of plasticity. Therefore, Lurcher mice cannot be considered as an exact model for CCAS of other etiologies. On the other hand, any effect to the cerebellar posterior lobe, notably lobule VI, lobule VII (including crus I and II of lobule VIIA, and lobule VIIB), and lobule IX leads to CCAS [11], as these lobes are interconnected to different cerebral areas [208]. In Lurcher mice, there is overall degradation of PCs, the only output of the cerebellar cortex, not restricted to some lobules only and being analogous to massive cerebellar decortications. Hence it leads to both motor and cognitive disorders. In CCAS, there is no correlation between motor deficit and cognitive performance and these functional impairments can occur separately in more restricted lesions of respective cerebellar parts [9]. Therefore, the hypothesis to be tested is whether Lurcher mice could be considered as a model for combined cerebellar motor syndrome and CCAS. Of course, the combination of both functional impairments brings methodological difficulties, since motor disabilities can influence performance in cognitive and behavioral tests, and vice versa, behavioral changes can modify the results of motor tests.

### Face Validity

The ***face validity*** is evaluated by how well a model replicates various signs of the disease, in this case, components of cerebellar syndromes.

Cerebellar motor syndrome is usually the most obvious and severe problem, reducing the quality of life in cerebellar patients. It consists of cerebellar ataxia, intention tremor, and muscle hypotonia called passivity. These basic components are then manifested with more specific disturbances, such as postural instability, gait disorders, dysarthria, handwriting changes and many others. Motor disorders, particularly cerebellar ataxia and equilibrium disorders are also well documented in cerebellar mutant mice, including Lurchers (Table 2). Naturally, for some specific signs, such as handwriting, there are no direct analogies between humans and mice. The comparability of others is problematic and limited. Examination of some of the specific signs is technically possible in mice, but has not been done in Lurchers, for example, gaze instability (described in hot-foot mice [209]) or vocalization recording and analysis (however, the question is whether analogy to dysarthria *sensu stricto* can be seen in mice, because of the substantially different vocalization patter in mice and humans). Gait analysis in Lurcher mice showed abnormalities similar to the human ataxic gait, such as shorter and irregular steps, inability to follow a straight direction of trajectory [120, 127–130]. The limiting factor, indeed, is the comparison of quadrupedal mice with bipedal humans. Nevertheless, on the general level, the basic features of motor disturbances in cerebellar patients and Lurcher mice are of the same nature (Table 2). Movements of Lurcher mice are ataxic, they show typical retropulsion and instability, which can be resemble that of human patients [121, 137, 139, 142–148]. Evaluation by EMG showed irregular muscle activities in both human patients and Lurcher mice [119, 129, 131, 132].

Cognitive and behavioral impairments are also observed in both Lurcher mice and CCAS patients (Table 3). Although comparing the symptoms in mice to human patients is incongruous, we highlight some analogies in the simple symptoms of CCAS patients and Lurcher mice (Table 3).

The main characteristic symptoms of patients with CCAS are executive, linguistic, visual-spatial, and neuropsychiatric impairments. Although not all symptoms occur in the same person, some of them are more prominent, depending on the different pathologies [5, 6, 9–12]. Executive functions include working memory, mental inflexibility, planning, multitasking, etc [6, 9]. Impairments in working memory in human patients (Schmahmann and Sherman 1998) can be compared to the impaired behavior of Lurcher mice in left-right discrimination in the T-maze compared to WT mice [168]. Failing in left-right discrimination in the T-maze [168], failing in the object localization task [171] and impaired learning in the radial maze [171] in Lurchers can also be compared to the inability of CCAS human patients in the verbal recall task to test short-term memory, where subjects were asked to recall the words learned previously [9]. The impairment of Lurcher mice in MWM [165–170], in spatial learning can be compared to the visual-spatial impairment of human CCAS patients [6, 10, 174]. Deficits in verbal associative learning, where CCAS subjects were asked to recall associated word pairs [9], can be compared to the slow response of Lurcher mice in the classical conditioning task [170]. Impairments in the mental inflexibility of CCAS patients [9] can be considered an analogy to the behavior inflexibility seen in Lurcher mice [175]. The attention deficit and hyperactivity of CCAS patients [6] resemble the increased spontaneous activity of Lurcher mice in the open field and hole-board tests [177, 179, 180]. Impairment in go/no-go task in CCAS subjects indicates deficits in sustained attention, impulse control, and disinhibition [9]. The latter in particular is typical for Lurcher mice [155, 170]. In addition, depression and anxiety have been seen in CCAS subjects [12, 178]. This is difficult to assess in Lurcher mice. Lack of immobility responses in the forced swimming test or analogous water conditions [180, 202] can be assigned as the absence of depressive-like behavior. Behavior in the elevated plus maze test and in the proximity of a predator resembles low anxiety [176, 177]. However, as discussed above, both these phenomena can be explained by behavioral disinhibition, and the high rise of corticosterone rather suggests (not confirms) stronger anxiety [155, 170, 203].

Cerebellar abnormalities have also been reported in several psychic disorders, such as autism spectrum disorders, schizophrenia or post-traumatic stress disorder [6, 9, 12, 210–214]. However, their relation to the cerebellum is not clear yet. Changes in cerebellar morphology or findings by functional imaging described in these diseases are often inconstant. In contrast, CCAS is primarily and directly defined as a neuropsychiatric manifestation of cerebellar damages that include significant cerebellar neuropathologies, although many individual components of CCAS are present among signs of other psychiatric syndromes [6, 9, 12]. Cerebellar mutant mice including Lurchers can be useful tools to investigate cerebellar involvement in the functional changes that are seen in those psychic diseases [175, 215–217]. Nevertheless, they definitely cannot be considered as models of schizophrenia or autism spectrum disorders as such.

### Predictive Validity

The ***predictive validity*** assesses whether a model accurately identifies a pharmacological (or other) treatment with equivalent clinical effectiveness, without making errors of omission or commission. There is a lack of studies testing experimental therapy in Lurcher mice comparable with clinical trials in human patients, because of poor evidence of its effect and/or safety. Therapy can be a symptomatic suppression of just the manifestations of the syndrome, or the causal treatment of the disease. Due to the rapid progress of degeneration, causal therapy would make sense only in newborn Lurcher mice, but is problematic in adult mice, unlike most of the late-onset human cerebellar degenerations.

Many of the studies on Lurcher mice were focused on motor performance. Rehabilitation is a standard and important component of therapy in ataxic patients [218, 219]. However, although Lurcher mice show surprisingly good motor learning ability [137, 151], enforced motor activity did not improve their performance in motor tasks involving different kinds of movements [115]. Neurotransplantation therapy, relatively intensively studied in Lurcher mice [114, 220–226], as well as pharmacotherapy showed rather inconsistent or pessimistic results regarding motor improvement [223], with the exception of mesenchymal stem cell grafting in newborn Lurchers reducing Purkinje cell loss [227].

Hitherto, we cannot consider that Lurcher mutant mice were clearly used to specifically test potential treatments for reducing cognitive and affective disorders associated with cerebellar pathologies. Only sparse studies mentioned that some molecules could, at least in part, reduce the emotional, cognitive and motor disorders in the Lurcher mutant. Benzodiazepine receptors were first investigated in Lurcher mutants [228, 229] and in other cerebellar mutants, such as reeler, staggerer and weaver (each of them having specific cerebellar degeneration) to depict the GABA binding sites in the cerebellum. Later, it was reported that i.p. chlordiazepoxide injection can drastically reduce behavioral disinhibition in the elevated plus maze, suggesting that this treatment could be useful for cerebellar patients. However, the authors also showed that, although the emotional behavior was normalized (mutant behaviors becoming similar to those of non-mutants in terms of open arms exploration), motor learning abilities were reduced in the Lurcher mice after chlordiazepoxide treatment [230]. Injection of amantadine and ketamine improved Lurchers’ motor coordination in the coat hanger test [231], and a low dose of delta-9-tetrahydrocannabinol enhanced motor performances and facilitated the acquisition of a new motor task of equilibrium in mutant mice, with complete normalization of motor coordination in the hole-board test at the dose of 4 mg/kg [232]. Although some authors recently proposed that cannabinoids and the endocannabinoid system are potential targets for the treatments of cerebellar symptoms [233, 234], there are no clinical trials permitting a conclusion of the benefits of these treatments. Dimethyl sulfoxide was also efficient to reduce memory impairments in the Morris water maze test, with a drastic decrease of motor activity (also observed in the open field test), suggesting a potential side effect of sedation and/or a possible role of the treatment on behavioral disinhibition [235]. Analogous pharmacotherapeutic studies in humans and Lurcher mice allowing direct comparison of the effects are lacking. In human cerebellar patients, various antidepressants were rather used to treat CCAS symptoms [236–239].

Another issue, indeed, is therapy of the underlying disease causing cerebellar function disorders. In this case, models of specific diseases defined by etiology are essential, since Lurcher mice are a model of a specific hereditary degeneration described in humans only in 2015 [240]. Therefore, other mouse models are preferred to test pharmacotherapy, experimental gene therapies, approaches based on antisense oligonucleotides, mainly genetically-engineered models of spinocerebellar ataxia or Friedreich’s ataxia [223, 241–248].

## Conclusion and Future Directions

A minor change in the complex brain can lead to enormous changes in the neuronal network. In Lurcher mice, olivocerebellar degradation causes various alterations in the underlying neuronal network, ultimately affecting their behavior. From the last four decades, a lot of research has been done on Lurcher mice to understand the impaired behavior and molecular changes. Nevertheless, many questions still need to be answered. The Lurcher mouse is a very good model for studying the effect of olivocerebellar degeneration and the impaired GluRδ2 receptor. Many disorders such as spinocerebellar ataxia type 18, intellectual disability, speech impairment, etc., are associated with the impaired GluRδ2 receptor in the cerebellum of human patients [209, 249, 250]. Studying the Lurcher model will give us insights into the proper function of the GluRδ2 receptor in the brain. The Lurcher mouse model, which also shows similarities with CCAS in cognitive impairment and visuomotor impairment, can be used to study disturbances of anatomical and functional connectivity of the cerebellum with the other brain areas.

As far as is known, neuropathology in heterozygous Lurcher mice consists selectively of olivocerebellar degeneration, although minor expression of the GluRδ2 and somewhat changed neurotransmitter levels were found elsewhere [105]. Therefore, these mice can be used to study the impacts of disorders in this system. In many cerebellar degenerations, neuropathology includes other brain regions as well and, therefore, association of certain functional impairments with certain structural damage is problematic. However, thorough assessment of the CNS of the Lurcher mice focused e.g., on potential mild morphological, cellular, biochemical changes, or gene expression are needed to verify whether the mutation does not primarily affect other structures. Furthermore, some disturbances may be due to secondary changes in function, or even the structure of areas synoptically (including indirect pathways) connected with the dysfunctional cerebellum.

Recently, in 2015, Coutelier et al. found human patients with congenital cerebellar ataxia with the same gain of function mutation in the GluRδ2 receptor as Lurcher mice, which also made them a human disease model [240]. Similarly, the null mutation in the same GluRδ2 receptor found in 2013 in human patients is mimicked by hot-foot cerebellar mutant mice [209, 251, 252].

Lurcher mice also serve as a tool to investigate therapeutic approaches to cerebellar degeneration [220–223, 227, 253]. As a model of selective olivocerebellar degeneration and complete functional cerebellar decortication, they can show whether a given therapy can improve the function of this system and thereby cerebellum-related signs of the disease. Since Lurcher mice combine motor, behavioral and cognitive pathological phenotypes, therapeutic studies can show selective or combined effects in only some, or in all, of these functional domains. Detailed knowledge of functional impairments in Lurcher mice and their mechanisms is therefore important for adequately designing the studies and correctly interpreting their results.

We propose that Lurcher mice suffering from early-onset olivocerebellar degeneration can be considered as a model to study the mechanisms of the components of both cerebellar motor syndrome and CCAS, with the limitation to analogous conditions (e.g., whole cerebellum affection, early loss of Purkinje cells) and of course with all the limitations of murine models of human diseases [254, 255].

## Data Availability

No datasets were generated or analysed during the current study.
